# Breastfeeding and maternal cardiovascular risk factors: 1982 Pelotas Birth Cohort

**DOI:** 10.1038/s41598-019-49576-1

**Published:** 2019-09-11

**Authors:** Natália P. Lima, Diego G. Bassani, Elma Izze S. Magalhães, Fernando C. Barros, Bernardo L. Horta

**Affiliations:** 1 Postgraduate Program in Epidemiology, Universidade Federal de Pelotas, Pelotas, Brazil; 2 Centre for Global Child Health, The Hospital for Sick Children, Toronto, Canada; 3 Department of Paediatrics and Dalla Lana School of Public Health, University of Toronto, Toronto, Canada; 4 Postgraduate Program in Health and Behavior, Universidade Católica de Pelotas, Pelotas, Rio Grande do Sul Brazil

**Keywords:** Epidemiology, Risk factors

## Abstract

This study evaluated the association of breastfeeding duration with maternal metabolic cardiovascular risk factors among women who have been prospectively followed since birth in a southern Brazilian city. In the unadjusted analysis, total cholesterol was higher among women who never breastfed in relation to those who breastfed ≥12 months. Among women with one livebirth, a shorter duration of breastfeeding was associated with lower HDL, while those with two or more livebirths and that breastfed for shorter time presented lower pulse wave velocity, glycaemia and non-HDL measures. After controlling for confounding variables, the magnitude of these associations decreased, and the confidence intervals included the reference. Concerning the duration of breastfeeding of the last child, the analysis was stratified by time since last birth. After controlling for confounders, systolic blood pressure was lower among women who breastfed 3 to <6 months and had a child within the last five years in relation to those who breastfed ≥6, but no clear trend was observed (p = 0.17). In conclusion, our findings suggest that there is no association between lactation and maternal cardiometabolic risk factors.

## Introduction

Beyond the known short^1^ and long-term benefits^2,3^ for the breastfed children, it has been reported that breastfeeding would be associated with maternal health, women who breastfeed have lower risk of breast cancer and higher birth spacing due to lactational amenorrhea^4^. It has been estimated that breastfeeding prevents about 20,000 deaths from breast cancer every year^5^. Additionally, it has been shown that long duration of breastfeeding reduces the risk of coronary heart disease^6,7^, type 2 diabetes^4,8–10^, whereas the evidence supporting the association with blood pressure^11–22^ and lipid profile^12,16,18,19,22,23^ are not clear.

Most of the studies on the long-term consequences of breastfeeding on maternal health have been carried out in high-income countries, where breastfeeding is positively associated with socioeconomic status^5^. Because cardiovascular risk factors are also associated with socioeconomic status^24–27^ and most studies adjusted the estimates for few socioeconomic confounders (*e*.*g*. only for schooling), perhaps not capturing its entire dimension, the possibility of residual confounding by socioeconomic status must be considered.

In the present study, we aimed at assessing the association of breastfeeding duration with maternal metabolic risk factors for cardiovascular disease among parous women who have been prospectively followed since birth in a southern Brazilian city, a setting where no strong social patterning of breastfeeding exists.

## Results

In 2012–13, when the participants were 29–31 years of age, we interviewed 1914 of the 2876 women born in 1982, which after taking into account the deaths identified among the cohort members, represented a follow-up rate of 71.0%. And 1147 had delivered at least one live birth and were not pregnant when interviewed, meeting the eligibility criteria. Information on breastfeeding duration and at least one of the metabolic cardiovascular risk factors was available for 1136 of the women eligible to enter the study.

Table 1 shows that 73.7% of the women included in the present study were white, mean proportion of European ancestry was 75%, and mean achieved schooling was 10.3 years. Over half of the women (52.7%) were primiparous and 45.6% had breastfed for at least 12 months (total number of months breastfeeding).

**Table 1:** Biological, socioeconomic, behavioral and health characteristics of the studied population (n = 1136). Pelotas, 2012.

Characteristics	N	%	Mean (SD)
Skin color			
White	837	73.7	—
Black	197	17.3	
Brown/Indigenous/Asian	102	9.0	
European ancestry	970	—	0.75 ± 0.20
Asset index^¥^			
D/E (poorest)	57	6.4	—
C	334	37.5	—
A/B (richest)	499	56.1	—
Years of schooling	1134	—	10.29 ± 3.96
Total duration of breastfeeding (months)*			
<1	124	10.9	—
1$\mbox{--}$<3	88	7.8	—
3–<6	142	12.5	—
6–<12	263	23.2	—
≥12	519	45.6	—
Parity			
1	599	52.7	—
2	336	29.6	—
≥3	201	17.7	—
Systolic blood pressure (mmHg)	1133	—	114.17 ± 12.13
Diastolic blood pressure (mmHg)	1133	—	73.44 ± 9.14
Carotid intima-media thickness (µm)	983	—	580.40 ± 16.22
Pulse wave velocity (m/s)	496	—	6.41 ± 1.05
Glycaemia (mg/dl)	1122	—	87.34 ± 22.91
Cholesterol (mg/dl)	1122	—	187.12 ± 35.39
LDL (mg/dl)	1122	—	107.03 ± 28.49
HDL (mg/dl)	1122	—	60.53 ± 12.74
Non-HDL (mg/dl)	1122	—	126.59 ± 32.83
Triglycerides (mg/dl)^#^	1122	—	90.13 ± 1.65

^¥^Brazilian Association of Research Companies. *Accumulated months of breastfeeding across all pregnancies resulting in ≥1 livebirth. ^#^Geometric mean.

Regarding the confounding variables, breastfeeding was higher in women with lower socioeconomic status in 2004–5. Systolic and diastolic blood pressure were negatively associated with family income in 2004–5, whereas carotid intima–media thickness was inversely associated with schooling (Supplementary Table S1). HDL cholesterol was directly associated with socioeconomic status (Supplementary Table S2).

The association between total duration of breastfeeding and blood pressure measures, carotid intima media-thickness and pulse wave velocity is presented in Table 2. In the analysis stratified by parity, in the crude analysis, women who breastfed 3 to <6 months and had had two or more livebirths showed lower pulse wave velocity than those who breastfed ≥12 months (β = −0.79, 95%CI: −1.4; −0.2, p = 0.04), but there was no clear pattern of association. After adjustment for the confounders, the magnitude of the estimative barely changed, but the confidence interval included the reference (β = −0.80, 95%CI: −1.7; 0.1, p-trend = 0.23). Blood pressure measures and carotid intima-media thickness did not present an association with breastfeeding. Additionally, no interaction between parity and breastfeeding was verified.

**Table 2:** Blood pressure, carotid intima-media thickness and pulse wave velocity according to total breastfeeding (n = 1136). Pelotas, 2012.

	N	Regression coefficient (95% confidence interval)							
		Systolic blood pressure (mmHg)		Diastolic blood pressure (mmHg)		Intima–media thickness (µm)		Pulse wave velocity (m/s)	
		Crude	Adjusted^†¥^	Crude	Adjusted^†¥^	Crude	Adjusted^†^	Crude	Adjusted^†^
Breastfeeding		p = 0.66*	p = 0.97*	p = 0.36*	p = 0.80*	p = 0.40*	p = 0.80*	p = 0.28*	p = 0.71*
Never	124	0.51 (−1.8; 2.8)	−0.05 (−2.8; 2.7)	0.80 (−0.9; 2.5)	0.27 (−1.8; 2.3)	1.41 (−1.9; 4.7)	−0.51 (−4.4; 3.4)	0.16 (−0.1; 0.5)	0.07 (−0.3; 0.5)
Ever	1012	Ref (0)	Ref (0)	Ref (0)	Ref (0)	Ref (0)	Ref (0)	Ref (0)	Ref (0)
Total sum of breastfeeding		p = 0.96**	p = 0.81**	p = 0.73**	p = 0.90*	p = 0.82**	P = 0.43*	p = 0.33*	p = 0.69*
Never	124	0.28 (−2.1; 2.7)	−0.28 (−3.2; 2.7)	0.64 (−1.2; 2.4)	0.10 (−2.0; 2.2)	1.23 (−2.2; 4.7)	−0.81 (−4.9; 3.3)	0.16 (−0.2; 0.5)	0.04 (−0.4; 0.5)
1–<3	88	−0.41 (−3.2; 2.3)	−0.17 (−4.0; 3.7)	−0.77 (−2.8; 1.3)	−0.71 (−3.5; 2.1)	−0.67 (−4.7; 3.3)	3.64 (−1.7; 9.0)	−0.06 (−0.4; 0.3)	−0.02 (−0.6; 0.5)
3–<6	142	−0.65 (−2.9; 1.6)	−0.50 (−3.3; 2.2)	0.21 (−1.5; 1.9)	0.28 (−1.7; 2.3)	−1.08 (−4.3; 2.2)	−0.73 (−4.5; 3.0)	−0.20 (−0.5; 0.1)	−0.24 (−0.6; 0.1)
6–<12	263	−0.40 (−2.2; 1.4)	−0.53 (−2.8; 1.8)	−0.48 (−1.8; 0.9)	−0.64 (−2.3; 1.0)	0.13 (−2.5; 2.7)	−1.72 (−4.9; 1.5)	0.11 (−0.1; 0.4)	0.06 (−0.3; 0.4)
≥12	519	Ref (0)	Ref (0)	Ref (0)	Ref (0)	Ref (0)	Ref (0)	Ref (0)	Ref (0)
Parity = 1		P = 0.91^‡^		P = 0.95^‡^		P = 0.42^‡^		P = 0.70^‡^	
Total sum of breastfeeding		P = 0.41*	p = 0.68*	P = 0.36*	p = 0.64**	P = 0.08**	P = 0.47**	P = 0.12**	P = 0.28**
Never	97	1.52 (−1.2; 4.2)	0.11 (−3.1; 3.4)	0.92 (−1.3; 3.1)	−0.19 (−2.7; 2.4)	3.40 (−0.6; 7.4)	0.79 (−4.2; 5.8)	0.35 (−0.1; 0.7)	0.28 (−0.2; 0.7)
1–<3	71	−1.65 (−4.7; 1.4)	−1.52 (−5.4; 2.4)	−1.83 (−4.3; 0.6)	−1.53 (−4.6; 1.5)	1.36 (−3.1; 5.8)	3.89 (−2.2; 9.9)	0.24 (−0.2; 0.7)	0.24 (−0.4; 0.8)
3–<6	108	−0.71 (−3.3; 1.9)	−1.94 (−5.0; 1.1)	−0.50 (−2.6; 1.6)	−1.10 (−3.5; 1.3)	−0.27 (−4.1; 3.5)	−1.46 (−6.1; 3.1)	0.21 (−0.2; 0.6)	0.08 (−0.4; 0.5)
6–<12	152	−0.18 (−2.6; 2.2)	−0.20 (−3.0; 2.6)	−0.55 (−2.5; 1.4)	−0.46 (−2.7; 1.7)	−0.71 (−4.1; 2.7)	−0.96 (−5.2; 3.3)	0.33 (−0.1; 0.7)	0.24 (−0.2; 0.6)
≥12	171	Ref (0)	Ref (0)	Ref (0)	Ref (0)	Ref (0)	Ref (0)	Ref (0)	Ref (0)
Parity > 1									
Total sum of breastfeeding		P = 0.25*	p = 0.21**	P = 0.56*	p = 0.34**	P = 0.48*	P = 0.41**	P = 0.04*	P = 0.23**
Never	27	−4.92 (−10.2; 0.3)	−4.05 (−10.6; 2.5)	−1.76 (−5.5; 2.0)	−1.95 (−6.4; 2.5)	−2.32 (−9.8; 5.2)	−4.92 (−13.7; 3.8)	0.55 (−0.3; 1.4)	−0.27 (−1.5; 1.0)
1–<3	17	3.94 (−2.6; 10.5)	−2.78 (−13.9; 8.3)	2.00 (−2.6; 6.6)	−3.33 (−11.0; 4.3)	−4.22 (−13.7; 5.3)	−1.01 (−16.9; 14.9)	−0.39 (−1.6; 0.8)	−0.35 (−2.8; 2.1)
3–<6	34	−1.08 (−5.9; 3.7)	0.19 (−5.9; 6.3)	1.31 (−2.1; 4.7)	1.44 (−2.8; 5.6)	0.24 (−6.7; 7.2)	3.46 (−4.7; 11.6)	−0.79 (−1.4; −0.2)	−0.80 (−1.7; 0.1)
6–<12	111	−0.90 (−3.8; 2.0)	−1.89 (−5.9; 2.1)	−0.77 (−2.8; 1.3)	−1.78 (−4.5; 0.9)	2.98 (−1.3; 7.2)	−2.33 (−7.5; 2.9)	0.07 (−0.3; 0.4)	0.04 (−0.6; 0.7)
≥12	348	Ref (0)	Ref (0)	Ref (0)	Ref (0)	Ref (0)	Ref (0)	Ref (0)	Ref (0)

^†^Adjusted for genomic ancestry, family income and maternal schooling at birth; asset index at childhood; and family income, schooling, asset index, energy intake, physical activity, alcohol consumption, current smoking and BMI at 2004–5. ^¥^Systolic and diastolic blood pressure were also adjusted for use of hypertensive drugs. *Test for heterogeneity. **Test for linear trend. ^‡^p-value for interaction.

Table 3 shows the association between total duration of breastfeeding and glycaemia and blood lipids. In the unadjusted analysis, total cholesterol was higher among women who never breastfed when comparing with those who breastfed for 12 months or longer (β = 7.17, 95%CI: 0.2; 14.2), but we did not observe a clear trend toward increasing total cholesterol, as duration of breastfeeding decreased (p-trend = 0.13). Furthermore, after controlling for confounders, the regression coefficient among those who never breastfed decreased from 7.17 to 2.27 (95%CI: −6.8; 11.3). Glycaemia and cholesterol fractions were not associated with breastfeeding and the magnitude of the regression coefficients decreased after controlling for confounding variables. On the other hand, when the analyses were stratified for parity, in the crude analysis, women who had had one livebirth and breastfed for 3–<6 showed lower HDL than those who breastfed ≥12 months (β = −3.16; 95%CI: −6.1; −0.2), although no linear association was observed (p = 0.11). But in the adjusted analysis, the regression coefficient reduced to −0.44 (95%CI: −4.1; 3.2). Among those women who had had two or more livebirths, glycaemia and non-HDL were lower in women with shorter duration of breastfeeding, than in those who breastfed 12 months or longer, but the associations did not show a clear pattern. After adjustment, glycaemia was lower among women who breastfed for 6 to <12 months (β = −5.47, 95%CI: −10.4; −0.5, p = 0.15), whereas for non-HDL, the crude analysis shown lower measure among those with 1 to < 3 months of breastfeeding (β = −17.3, 95%CI: −33.5; −1.2), but the confidence interval included the reference after adjustment and no association was observed (p = 0.12). Also, there was no significant interaction between breastfeeding and parity.

**Table 3:** Glycaemia and lipid profile according to total breastfeeding (n = 1136). Pelotas, 2012.

	N	Regression coefficient (95% confidence interval)											
		Glycaemia (mg/dl)		Cholesterol (mg/dl)		LDL (mg/dl)		HDL (mg/dl)		Non-HDL (mg/dl)		Triglycerides^#^ (mg/dl)	
		Crude	Adjusted^†¥^	Crude	Adjusted^†^	Crude	Adjusted^†^	Crude	Adjusted^†^	Crude	Adjusted^†^	Crude	Adjusted^†^
Breastfeeding		p = 0.50*	p = 0.95*	p = 0.07*	p = 0.96*	p = 0.13*	p = 0.83*	p = 0.09*	p = 0.24*	p = 0.19*	p = 0.70*	p = 0.59*	p = 0.42*
Never	124	−1.48 (−5.8; 2.8)	−0.15 (−4.8; 4.5)	6.18 (−0.5; 12.8)	0.20 (−8.4; 8.8)	4.17 (−1.2; 9.5)	−0.78 (−7.8; 6.3)	2.06 (−0.3; 4.5)	1.73 (−1.2; 4.6)	4.12 (−2.1; 10.3)	−1.53 (−9.4; 6.4)	1.03 (0.9; 1.1)	0.95 (0.8; 1.1)
Ever	1012	Ref (0)	Ref (0)	Ref (0)	Ref (0)	Ref (0)	Ref (0)	Ref (0)	Ref (0)	Ref (0)	Ref (0)	Ref (1)	Ref (1)
Total sum of breastfeeding		p = 0.56*	p = 0.92**	p = 0.13**	p = 0.45**	p = 0.22*	p = 0.53*	p = 0.04**	p = 0.11**	p = 0.21*	P = 0.66*	p = 0.45**	p = 0.53*
Never	124	−1.10 (−5.6; 3.4)	−0.40 (−5.3; 4.5)	7.17 (0.2; 14.2)	2.27 (−6.8; 11.3)	4.66 (−1.0; 10.3)	0.83 (−6.6; 8.3)	2.41 (−0.1; 4.9)	2.08 (−1.0; 5.2)	4.76 (−1.7; 11.2)	0.19 (−8.1; 8.5)	1.04 (0.9; 1.1)	0.98 (0.9; 1.1)
1–<3	88	0.64 (−4.6; 5.9)	0.50 (−5.8; 6.8)	−2.08 (−10.1; 6.0)	2.58 (−9.1; 14.2)	−3.66 (−10.1; 2.8)	−1.60 (−11.1; 7.9)	2.56 (−0.3; 5.5)	2.85 (−1.1; 6.8)	−4.64 (−12.1; 2.8)	−0.26 (−11.0; 10.4)	0.99 (0.9; 1.1)	1.10 (0.9; 1.3)
3–<6	142	3.15 (−1.1; 7.4)	−0.60 (−5.2; 4.0)	3.35 (−3.3; 10.0)	5.02 (−3.4; 13.4)	3.01 (−2.3; 8.3)	3.98 (−2.9; 10.9)	−0.32 (−2.7; 2.1)	0.27 (−2.6; 3.1)	3.67 (−2.4; 9.8)	4.75 (−3.0; 12.5)	1.04 (0.9; 1.1)	1.07 (0.9; 1.2)
6–<12	263	−0.43 (−3.9; 3.0)	−0.76 (−4.6; 3.1)	2.69 (−2.6; 8.0)	4.04 (−3.0; 11.1)	1.45 (−2.8; 5.7)	4.13 (−1.7; 9.9)	0.67 (−1.2; 2.6)	0.37 (−2.0; 2.8)	2.02 (−2.9; 6.9)	3.67 (−2.8; 10.2)	1.03 (0.9; 1.1)	1.01 (0.9; 1.1)
≥12	519	Ref (0)	Ref (0)	Ref (0)	Ref (0)	Ref (0)	Ref (0)	Ref (0)	Ref (0)	Ref (0)	Ref (0)	Ref (1)	Ref (1)
Parity = 1		P = 0.91^‡^		P = 0.61^‡^		P = 0.85^‡^		P = 0.52^‡^		P = 0.82^‡^		P = 0.31^‡^	
Total sum of breastfeeding		P = 0.60*	p = 0.73*	P = 0.34*	P = 0.46**	P = 0.47**	P = 0.87*	P = 0.11*	P = 0.28**	P = 0.35**	P = 0.68**	P = 0.18**	P = 0.25*
Never	97	0.06 (−6.2; 6.3)	1.03 (−4.8; 6.9)	6.62 (−2.1; 15.3)	5.64 (−6.1; 17.4)	4.87 (−2.3; 12.0)	1.98 (−7.6; 11.6)	0.52 (−2.6; 3.6)	2.47 (−1.5; 6.4)	6.10 (−2.1; 14.3)	3.17 (−7.7; 14.0)	1.08 (0.9; 1.2)	1.03 (0.9; 1.2)
1–<3	71	2.05 (−4.9; 9.0)	2.56 (−4.4; 9.5)	−3.08 (−12.7; 6.6)	5.36 (−8.6; 19.3)	−2.94 (−10.9; 5.0)	−0.10 (−11.5; 11.3)	−0.85 (−4.3; 2.6)	2.08 (−2.6; 6.8)	−2.23 (−11.3; 6.8)	3.28 (−9.6; 16.1)	1.08 (0.9; 1.2)	1.20 (0.9; 1.5)
3–<6	108	4.70 (−1.3; 10.7)	−0.44 (−5.9; 5.0)	−1.23 (−9.6; 7.2)	2.05 (−8.9; 13.0)	0.56 (−6.3; 7.4)	1.16 (−7.8; 10.1)	−3.16 (−6.1; −0.2)	−0.44 (−4.1; 3.2)	1.93 (−5.9; 9.8)	2.48 (−7.6; 12.6)	1.08 (0.9; 1.2)	1.12 (0.9; 1.3)
6–<12	152	1.23 (−4.2; 6.7)	2.81 (−2.1; 7.8)	2.91 (−4.7; 10.5)	7.68 (−2.3; 17.6)	1.35 (−4.9; 7.6)	4.22 (−3.9; 12.4)	0.71 (−2.0; 3.4)	1.73 (−1.6; 5.1)	2.20 (−4.9; 9.3)	5.95 (−3.2; 15.1)	1.06 (0.9; 1.2)	1.12 (0.9; 1.3)
≥12	171	Ref (0)	Ref (0)	Ref (0)	Ref (0)	Ref (0)	Ref (0)	Ref (0)	Ref (0)	Ref (0)	Ref (0)	Ref (1)	Ref (1)
Parity > 1													
Total sum of breastfeeding		P = 0.32**	p = 0.15*	P = 0.43*	P = 0.18**	P = 0.29*	P = 0.20*	P = 0.23*	P = 0.73*	P = 0.17*	P = 0.12*	P = 0.34**	P = 0.42**
Never	27	−3.31 (−11.4; 4.7)	−2.64 (−10.7; 5.4)	−3.66 (−17.7; 10.4)	−15.83 (−33.0; 1.4)	−0.63 (−11.8; 10.6)	−11.34 (−25.6; 3.0)	−1.43 (−6.4; 3.5)	−0.96 (−6.9; 5.0)	−2.23 (−15.2; 10.8)	−14.87 (−30.7; 1.0)	0.97 (0.8; 1.2)	0.90 (0.7; 1.1)
1–<3	17	−2.95 (−12.9; 7.0)	−7.55 (−21.2; 6.1)	−13.27 (−30.7; 4.2)	−13.80 (−43.1; 15.5)	−12.10 (−26.0; 1.8)	−14.17 (−38.5; 10.2)	4.03 (−2.1; 10.2)	4.04 (−6.0; 14.1)	−17.3 (−33.5; −1.2)	−17.84 (−44.8; 9.1)	0.80 (0.6; 1.0)	0.94 (0.6; 1.4)
3–<6	34	−0.10 (−7.3; 7.1)	2.29 (−5.4; 10.0)	6.56 (−6.1; 19.2)	10.17 (−6.0; 26.3)	6.72 (−3.3; 16.8)	7.27 (−6.1; 20.7)	−0.61 (−5.1; 3.8)	0.39 (−5.2; 5.9)	7.17 (−4.5; 18.9)	9.78 (−5.1; 24.6)	0.99 (0.8; 1.2)	1.08 (0.9; 1.3)
6–<12	111	−2.20 (−6.6; 2.2)	−5.47 (−10.4; −0.5)	−1.40 (−9.1; 6.3)	−1.33 (−11.9; 9.2)	0.24 (−5.9; 6.4)	3.48 (−5.3; 12.2)	−2.51 (−5.2; 0.2)	−1.93 (−5.6; 1.7)	1.10 (−6.0; 8.3)	0.60 (−9.1; 10.3)	0.99 (0.9; 1.1)	0.91 (0.8; 1.1)
≥12	348	Ref (0)	Ref (0)	Ref (0)	Ref (0)	Ref (0)	Ref (0)	Ref (0)	Ref (0)	Ref (0)	Ref (0)	Ref (1)	Ref (1)

^#^Log-transformed data (multiplicative effect). ^†^Adjusted for genomic ancestry, family income and maternal schooling at birth; asset index at childhood; and family income, schooling, asset index, energy intake, physical activity, alcohol consumption, current smoking and BMI at 2004–5. ^¥^Glycaemia was also adjusted for use of hypoglycemic drugs. *Test for heterogeneity. **Test for linear trend. ^‡^p-value for interaction.

Tables 4 and 5 show the results of the analyses restricted to duration of breastfeeding of the last child, after adjusting for confounders. These analyses were stratified for time since last birth. Systolic blood pressure was lower among women who breastfed 3 to <6 months and had a child within the last five years when compared with those who breastfed ≥6 (β = −3.36, 95%CI: −6.7; −0.5), though no clear trend was observed (p = 0.17). We did not observe association in those whose last childbirth was ≥5 years and there was no interaction (p-interaction = 0.13). Regarding the other cardiovascular risk factors, we did not observe association with the last child breastfeeding.

**Table 4:** Blood pressure, carotid intima-media thickness and pulse wave velocity according to last child breastfeeding and stratified to time since last birth (n = 1133). Pelotas, 2012.

	N	Adjusted regression coefficient (95% confidence interval)†			
		Systolic blood pressure^¥^ (mmHg)	Diastolic blood pressure (mmHg)	Intima–media thickness (µm)	Pulse wave velocity (m/s)
**Last child breastfeeding**					
*Time since last birth*		P = 0.21^‡^	P = 0.07^‡^	P = 0.25^‡^	P = 0.86^‡^
<*5 years*		p = 0.34*	p = 0.09*	P = 0.10*	P = 0.25*
Never	68	1.82 (−1.9; 5.5)	2.26 (−0.4; 4.9)	−3.90 (−8.6; 0.8)	0.32 (−0.2; 0.9)
Ever	489	Ref (0)	Ref (0)	Ref (0)	Ref (0)
≥*5 years*		p = 0.59*	p = 0.55*	P = 0.86*	P = 0.79*
Never	84	−1.04 (−4.8; 2.7)	−0.86 (−3.7; 1.9)	0.53 (−5.3; 6.4)	0.07 (−0.4; 0.6)
Ever	492	Ref (0)	Ref (0)	Ref (0)	Ref (0)
**Total last child breastfeeding (months)**					
*Time since last birth*		P = 0.13^‡^	P = 0.15^‡^	P = 0.24^‡^	P = 0.95^‡^
<*5 years*		p = 0.17*	p = 0.21*	P = 0.16*	P = 0.47**
Never	68	1.02 (−2.8; 4.8)	1.88 (−0.8; 4.6)	−3.51 (−8.3; 1.3)	0.28 (−0.3; 0.9)
1–<3	66	−1.44 (−5.5; 2.6)	−1.68 (−4.6; 1.2)	4.16 (−1.2; 9.5)	−0.08 (−0.8; 0.7)
3–<6	95	−3.36 (−6.7; −0.5)	−1.06 (−3.4; 1.3)	−0.17 (−4.4; 4.0)	−0.15 (−0.7; 0.4)
≥6	328	Ref (0)	Ref (0)	Ref (0)	Ref (0)
≥*5 years*		P = 0.28**	p = 0.28**	P = 0.57*	P = 0.64**
Never	84	−1.87 (−5.8; 2.1)	−1.37 (−4.3; 1.6)	0.65 (−5.4; 6.7)	0.07 (−0.5; 0.6)
1–<3	80	−1.48 (−5.8; 2.9)	−1.10 (−4.3; 2.1)	3.68 (−2.8; 10.2)	0.22 (−0.4; 0.8)
3–<6	106	−2.41 (−5.9; 1.1)	−1.41 (−4.0; 1.2)	−1.57 (−6.8; 3.6)	−0.09 (−0.5; 0.3)
≥6	306	Ref (0)	Ref (0)	Ref (0)	Ref (0)

^†^Adjusted for genomic ancestry, family income and maternal schooling at birth; asset index at childhood; family income, schooling, asset index, energy intake, physical activity, alcohol consumption, current smoking and BMI at 2004–5; and parity. ^¥^Systolic and diastolic blood pressure were also adjusted for use of hypertensive drugs. ^‡^p-value for interaction. ^*^Test for heterogeneity. ^**^Test for linear trend.

**Table 5:** Glycaemia and lipid profile according to last child breastfeeding and stratified to time since last birth (n = 1133). Pelotas, 2012.

	N	Adjusted regression coefficient (95% confidence interval)^†^					
		Glycaemia^¥^ (mg/dl)	Cholesterol (mg/dl)	LDL (mg/dl)	HDL (mg/dl)	Non-HDL (mg/dl)	Triglycerides^#^ (mg/dl)
**Last child breastfeeding**							
*Time since last birth*		P = 0.75^‡^	P = 0.67^‡^	P = 0.44^‡^	P = 0.77^‡^	P = 0.57^‡^	P = 0.86^‡^
<*5 years*		p = 0.66*	P = 0.88*	P = 0.82*	P = 0.65*	P = 0.99*	P = 0.73*
Never	68	−1.37 (−7.4; 4.7)	0.88 (−10.7; 12.5)	1.14 (−8.6; 10.8)	0.89 (−2.9; 4.7)	−0.01 (−10.9; 10.9)	0.97 (0.8; 1.2)
Ever	489	Ref (0)	Ref (0)	Ref (0)	Ref (0)	Ref (0)	Ref (1)
≥*5 years*		p = 0.66*	P = 0.87*	P = 0.53*	P = 0.19*	P = 0.49*	P = 0.40*
Never	84	−1.20 (−6.5; 4.1)	−0.91 (−12.2; 10.4)	−2.87 (−11.9; 6.2)	2.64 (−1.3; 6.6)	−3.55 (−13.6; 6.5)	0.94 (0.8; 1.1)
Ever	492	Ref (0)	Ref (0)	Ref (0)	Ref (0)	Ref (0)	Ref (1)
**Total last child breastfeeding (months)**							
*Time since last birth*		P = 0.67^‡^	P = 0.51^‡^	P = 0.83^‡^	P = 0.19^‡^	P = 0.83^‡^	P = 0.74^‡^
<*5 years*		p = 0.34**	P = 0.32**	P = 0.52**	P = 0.15**	P = 0.58**	P = 0.73*
Never	68	−2.11 (−8.4; 4.1)	2.77 (−9.1; 14.7)	1.98 (−8.0; 12.0)	1.84 (−2.1; 5.7)	0.94 (−10.3; 12.2)	0.98 (0.8; 1.2)
1–<3	66	−2.89 (−9.7; 3.9)	8.45 (−4.5; 21.4)	3.66 (−7.2; 14.5)	2.51 (−1.7; 6.7)	5.93 (−6.3; 18.1)	1.11 (0.9; 1.3)
3–<6	95	−2.17 (−7.7; 3.4)	4.76 (−5.8; 15.3)	2.15 (−6.7; 11.0)	3.46 (0.1; 6.9)	1.30 (−8.7; 11.3)	1.00 (0.9; 1.2)
≥6	328	Ref (0)	Ref (0)	Ref (0)	Ref (0)	Ref (0)	Ref (1)
≥*5 years*		p = 0.70**	P = 0.92**	P = 0.70*	P = 0.33*	P = 0.81*	P = 0.18*
Never	84	−1.36 (−6.9; 4.2)	−0.38 (−12.2; 11.4)	−1.87 (−11.4; 7.6)	2.12 (−2.0; 6.2)	−2.50 (−13.0; 8.1)	0.95 (0.8; 1.1)
1–<3	80	0.23 (−5.9; 6.3)	2.44 (−10.5; 15.3)	−0.38 (−10.7; 10.0)	0.27 (−4.2; 4.7)	2.18 (−9.4; 13.7)	1.17 (0.9; 1.4)
3–<6	106	−0.72 (−5.6; 4.1)	0.83 (−9.4; 11.1)	4.11 (−4.1; 12.4)	−2.21 (−5.8; 1.3)	3.04 (−6.1; 12.2)	0.97 (0.9; 1.1)
≥6	306	Ref (0)	Ref (0)	Ref (0)	Ref (0)	Ref (0)	Ref (1)

^#^Log-transformed data (multiplicative effect). ^†^Adjusted for genomic ancestry, family income and maternal schooling at birth; asset index at childhood; family income, schooling, asset index, energy intake, physical activity, alcohol consumption, current smoking and BMI at 2004–5; and parity. ^¥^Glycaemia was also adjusted for use of hypoglycemic drugs. ^‡^p-value for interaction. ^*^Test for heterogeneity. ^**^Test for linear trend.

## Discussion

In a population that has been prospectively followed since birth, after controlling for biological, socioeconomic and behavioral variables, no association between breastfeeding and maternal cardiometabolic risk factors was observed.

Observational studies evaluating the association of breastfeeding with maternal health outcomes have reported benefits on metabolic risk factors, such as blood pressure^11,13–16,18–22^, glucose^9,10,16,19,21,22,28–30^ and lipids^12,16,19,22^, whereas others have not observed such associations^12,18,23^. Concerning the carotid intima-media thickness, one study showed a positive association^31^ and two others did not observe such association^32,33^, while for pulse wave velocity one study did not found an association^32^. As mentioned before, most of the published studies have been carried out in high income countries, where breastfeeding is positively associated with socioeconomic status^5^, and adjusted for few socioeconomic variables. In this context, the estimates could be biased by residual confounding by socioeconomic status. In our study, breastfeeding was inversely associated with socioeconomic status, and we adjusted the estimates for several socioeconomic factors to minimize the possibility of residual confounding. Therefore, our results were probably not due to residual confounding. However, considering the natural history of the risk factors evaluated, our studied population is young, and these morbidities may not yet be clinically detectable, which may explain in part our negative findings.

Studies have evaluated weather time since last childbirth modifies the association of breastfeeding with cardiovascular risk factors. Gunderson *et al*., using data from the Coronary Artery Risk Development in Young Adults, assessed the effect of breastfeeding on subclinical atherosclerosis and observed no interaction (p ≥ 0.1)^31^. Stuebe *et al*. also reported a non-significant interaction with time since last birth when evaluating parous women from the Nurses’ Health Study (NHS) and Nurses’ Health Study II (NHS II) (NHS: p = 0.32; NHS II: p = 0.54), despite having observed association between breastfeeding and type II diabetes in women who gave birth in the past 15 years in both NHS and NHSII and no association in those with time higher than 15 in the NHS II and a reduced association in the NHS^29^. When assessing the association of breastfeeding and maternal cardiovascular disease in postmenopausal women from the Women’s Health Initiative, Schwarz *et al*. verified a significative interaction with age (p = 0.02), but no with age at last lactation (p = 0.58)^19^. In addition, other studies showed that the association of breastfeeding with metabolic risk factors was weaker in women with longer time since last birth^9,30^ and declined as age increased^15,16,34,35^; but in the latter the effect modification may be due to time since last birth, and not properly due to age.

One limitation of observational studies is that residual confounding may bias the estimates. In contrast, exchangeability between the comparison groups is expected in experimental studies. Oken *et al*.^17^ evaluated the effect of breastfeeding on maternal blood pressure using data from the Promotion of Breastfeeding Intervention Trial (PROBIT), in which hospitals and polyclinics from Belarus were randomized to implement the Baby-Friendly Hospital Initiative. On intention-to-treat analysis, they observed lower levels of systolic (mean difference: −0.81, 95%CI: −3.33; 1.71) and diastolic blood pressure (mean difference: −1.09, 95%CI: −2.43; 0.25) and odds of hypertension (OR: 0.85, 95%CI: 0.64; 1.12) in the intervention arm compared to the control, but these associations were not statistical significant. However, one limitation of this study is that the compliance was low, that is women from the intervention group did not breastfeed their child while some from the control group breastfed, which reduced the power to detect differences between the groups. Therefore, the non-statistically significant association may not be due to the nonexistence of an association.

Our study has several strengths. It was based on information from a large birth cohort with a high follow-up rate. We were able to evaluate the association of breastfeeding with several metabolic risk factors and all measures were obtained by trained interviewers. Also, the information on confounding factors was collected prospectively, reducing the chance of residual confounding. Although the attrition rate was slightly higher among those in the extreme socioeconomic categories, breastfeeding per livebirth was independent of socioeconomic status, so this small difference is unlikely of having introduced a selection bias. However, some limitations must be considered. We were not able to adjust for some possible confounding factors, such as pre-gestational body mass index and weight gain during pregnancy, because we did not collect information on these variables. For the same reason, we could not adjust for smoking or household exposure to tobacco in pregnancy. When controlling the analysis for tobacco smoking in the 2004–5 as a proxy for those variables, the magnitude of the differences barely decreased. As people hardly quit smoking due to addiction, and it is not expected that non-smokers women start the habit during pregnancy, we believe it is not likely that our results are due to confounding for tobacco in pregnancy. We do not have data on patterns and daily frequency of lactation, and also could not distinguish between direct breastfeeding and pumped breast milk, so our findings should be interpreted with caution. Additionally, glycaemia was evaluated using non-fasting blood glucose measure. Even having adjusted for time since last meal, it may have introduced a non-differential misclassification, but the magnitude of the association was small. Therefore, it is unlikely that this negative result was due to the misclassification.

In conclusion, our findings suggest that there is no association between lactation and maternal cardiometabolic risk factors.

## Materials and Methods

### Participants

This study is based on data from the 1982 Pelotas Birth Cohort Study. In 1982, all the maternity hospitals located in Pelotas, a southern Brazilian city, were visited daily and all births identified (n = 7392). Based on data from birth registration and a city census, we estimated that our hospital sample accounts for 99.2% of all births in the city. Those live births whose family lived in the urban area of the city were examined and their mothers interviewed soon after delivery (n = 5914). These subjects have been prospectively followed at different ages. Further details on the study methodology have been published elsewhere^36,37^. From June 2012 to February 2013, we tried to follow the whole cohort. Multiple strategies were used to locate the study participants, who were invited to attend the research clinic to be interviewed, examined, and provided a blood sample^38^. Of the 5914 members of the 1982 Cohort, 3701 agreed to participate, which added to the 325 deaths identified among the cohort participants, represented a follow-up rate of 68.1%. In the present study we included only women evaluated in 2012–13, who had a previous delivery, and were not pregnant when interviewed (n = 1147).

### Exposure

In 2012–13 visit, the subjects provided the following information on each offspring: birthweight, type of delivery and duration of breastfeeding. Cumulative lifetime duration of breastfeeding (in months) was obtained by summing the duration of lactation of all offspring. We also assess the duration of breastfeeding of the last child.

### Outcomes

In the present study, we evaluated the following outcomes that were evaluated in the 2012–13 visit:

- Blood pressure was measured twice, at the beginning and at the end of the anthropometrical assessment, on the left arm, using an automatic digital sphygmomanometer, model Omron HEM 705CPINT, with a specific cuff for obese individuals. The mean of the measurements was used in the analysis.
- Carotid intima-media thickness of the posterior wall of right and left carotid arteries was evaluated in longitudinal planes using a Toshiba Xario ultrasound^39^. A 10 mm-long section of the common carotid artery was imaged proximal to carotid bulb using the Carotid Analyzer for Research, Medical Imaging Application-LLC, that evaluated the arithmetic mean of 90 frames. The mean of the measurements was used in the analysis.
- Pulse wave velocity was assessed using a portable ultrasound, Sphygmocor® (AtCor Medical, Version 9.0, Sydney, Australia) in supine position, after resting for 5 minutes. Pulse wave velocity was estimated by dividing the distance between carotid and femoral sites by the transit time between the carotid and femoral pulse wave.
- Random blood glucose was measured using an automatic enzymatic colorimetric method, BS-380, Mindray (Shenzhen Mindray Bio-Medical Electronics Co., Ltd, China), and the assay sensitivity was 1.31 mg/dl. Because glucose levels vary according to fasting time, estimates were adjusted for time since the last meal^40^.
- HDL, LDL, total cholesterol and triglycerides were measured using an enzymatic assay (Shenzhen Mindray Bio-Medical Electronics Co., Ltd, China), and the assays sensitivity was 2.9996 mg/dl, 0.2540 mg/dl, 1.472 mg/dl and 2.845 mg/dl, respectively. Non-HDL was obtained by subtracting HDL from total cholesterol.

### Confounders

The following variables were considered as possible confounders. Family income in minimum wages and maternal schooling (in complete years of schooling) at birth were provided by the mother in the perinatal study. A household asset index in childhood was estimated using principal component analysis and based on household characteristics, such as type of building, piped water in the household, type of lavatory, presence of a gas stove at home, wood stove at home and number of bedrooms. Other variables collected during the 2004–5 follow-up visits were used in the analysis, such as family income (in Brazilian reais), schooling, asset index according to criteria of the Brazilian Association of Research Companies, European genomic ancestry (based on approximately 370,000 SNPs mutually available for the Pelotas cohort and selected samples of the HapMap and Human Genome Diversity - ADMIXTURE was used to estimate the genomic ancestry of each subject)^41^, daily energy intake based on a food frequency questionnaire with recall period of 12 months, leisure time physical activity assessed through the International Physical Activity Questionnaire (minutes/week)^42^, alcohol consumption in the last week, self-reported tobacco smoking and body mass index. When evaluating systolic and diastolic blood pressure and glycaemia, we also adjusted for treatment with antihypertensive and hypoglycemic drugs, respectively.

### Statistical analyses

Analysis of variance (ANOVA) and chi-square test were used to assess differences between means and proportions, respectively. Because all the outcomes were continuous, linear regression was used to evaluate the association of breastfeeding duration with maternal metabolic cardiovascular risk factors. Triglycerides were log-transformed because its distribution was asymmetric. Statistical comparisons between groups were based on tests of heterogeneity and linear trend, and the one with the lower p-value was presented. In the multivariable analysis, estimates were adjusted for biological, socioeconomic and behavioral variables. The analysis was also stratified for parity. When evaluating the last child breastfeeding, we adjusted for parity and stratified the analysis by time since last birth. We used Stata 13.0 for the analyses.

### Ethics statement

All participants signed a written informed consent and the Research Ethics Committee of the Faculty of Medicine, Federal University of Pelotas, approved the study protocol (protocol number: Of.16/12). All methods were performed in accordance with relevant guidelines and regulations.

## Supplementary information

Supplementary Table S1. Duration of breastfeeding, blood pressure, carotid intima-media thickness and pulse wave velocity according to biological, socioeconomic and behavioral variables (n=1136). Pelo

Supplementary Table S2. Glycaemia and lipid profile according to biological, socioeconomic and behavioral variables (n=1136). Pelotas, 1982–2012.

## Data Availability

The dataset is available from the corresponding author on reasonable request.
