# Supplementary material for: Breastfeeding and maternal cardiovascular risk factors: 1982 Pelotas Birth Cohort
Source: Sci Rep. 2019 Sep 11;9:13092. doi: 10.1038/s41598-019-49576-1 (PMC6739402; doi:10.1038/s41598-019-49576-1)
Supplement: Supplementary file 1 — Supplementary Table S1. Duration of breastfeeding, blood pressure, carotid intima-media thickness and pulse wave velocity according to biological, socioeconomic and behavioral variables (n=1136). Pelo [file 41598_2019_49576_MOESM1_ESM.doc]

**Breastfeeding and maternal cardiovascular risk factors: 1982 Pelotas Birth Cohort**

**Authors**

Natália P. Lima

Diego G. Bassani

Elma Izze S. Magalhães

Fernando C. Barros

Bernardo L. Horta

| **Supplementary Table S1.** Duration of breastfeeding, blood pressure, carotid intima-media thickness and pulse wave velocity according to biological, socioeconomic and behavioral variables (n=1136). Pelotas, 1982-2012. | | | | | | | | | | |
| --- | --- | --- | --- | --- | --- | --- | --- | --- | --- | --- |
| Variables | Total sum of breastfeeding (months) | | | | | | Systolic blood pressure (mmHg) | Diastolic blood pressure (mmHg) | Carotid intima–media thickness (µm) | Pulse wave velocity (m/s) |
|  | Never | 1 - <3 | 3 - <6 | 6 - <12 | | ≤12 |  |  |  |  |
|  | N (%) | N (%) | N (%) | N (%) | | N (%) | Mean (CI95%) | Mean (CI95%) | Mean (CI95%) | Mean (CI95%) |
| **Birth** |  |  |  |  | |  |  |  |  |  |
| European ancestry (quintiles) | p=0.16* | | | | | | p<0.01** | p<0.01** | p=0.15** | p=0.73** |
| First | 23 (11.9) | 13 (6.7) | 18 (9.3) | 44 (22.7) | 96 (49.4) | | 117.8 (115.9; 119.8) | 75.9 (74.5; 77.4) | 582.6 (579.8; 585.4) | 6.5 (6.3; 6.7) |
| Second | 16 (8.3) | 15 (7.7) | 19 (9.8) | 43 (22.2) | 101 (52.0) | | 112.7 (110.8; 114.6) | 72.4 (71.1; 73.7) | 580.7 (577.6; 583.8) | 6.5 (6.2; 6.7) |
| Third | 22 (11.3) | 12 (6.2) | 25 (12.9) | 45 (23.2) | 90 (46.4) | | 113.6 (112.0; 115.2) | 73.3 (72.1; 74.6) | 580.8 (578.6; 583.1) | 6.5 (6.2; 6.7) |
| Fourth | 27 (13.9) | 20 (10.3) | 22 (11.3) | 42 (21.7) | 83 (42.8) | | 113.8 (112.2; 115.4) | 73.3 (72.0; 74.6) | 580.2 (577.7; 582.8) | 6.4 (6.1; 6.6) |
| Fifth | 26 (13.4) | 15 (7.7) | 36 (18.6) | 47 (24.2) | 70 (36.1) | | 114.2 (112.6; 115.9) | 73.2 (71.9; 74.5) | 577.9 (575.9; 580.0) | 6.3 (6.1; 6.5) |
| Family income|| | p=0.08* | | | | | | p=0.27** | p=0.49** | p=0.86** | p=0.17** |
| ≤1 | 28 (9.9) | 21 (7.4) | 22 (7.8) | 62 (21.9) | 150 (53.0) | | 114.7 (113.0; 116.4) | 74.1 (72.9; 75.3) | 580.8 (578.7; 582.8) | 6.4 (6.2; 6.6) |
| 1.1-3 | 69 (11.0) | 45 (7.2) | 93 (14.9) | 137 (21.9) | 281 (45.0) | | 114.4 (113.5; 115.3) | 73.3 (72.6; 74.0) | 580.5 (579.1; 582.0) | 6.4 (6.3; 6.5) |
| 3.1-6 | 19 (11.5) | 15 (9.0) | 21 (12.7) | 43 (25.9) | 68 (40.9) | | 113.1 (111.6; 114.7) | 73.2 (72.0; 74.4) | 579.9 (577.1; 582.7) | 6.5 (6.3; 6.8) |
| 6.1-10 | 4 (10.3) | 3 (7.7) | 3 (7.7) | 15 (38.5) | 14 (35.8) | | 113.2 (109.3; 117.2) | 72.1 (69.2; 75.1) | 577.7 (574.8; 580.7) | 6.4 (5.9; 6.9) |
| >10 | 4 (23.5) | 2 (11.8) | 2 (11.8) | 5 (29.4) | 4 (23.5) | | 109.1 (104.7; 113.4) | 71.3 (67.6; 75.1) | 580.1 (577.0; 583.2) | 5.6 (4.2; 7.0) |
| Maternal schooling | p=0.35* | | | | | | p=0.05** | p=0.42** | p=0.56** | p=0.25** |
| 0-4 | 40 (9.0) | 37 (8.3) | 54 (12.1) | 100 (22.4) | 216 (48.2) | | 114.1 (112.9; 115.3) | 73.4 (72.6; 74.3) | 581.1 (579.2; 582.9) | 6.4 (6.3; 6.6) |
| 5-8 | 65 (12.8) | 39 (7.7) | 70 (13.7) | 110 (21.6) | 226 (44.2) | | 114.4 (113.4; 115.4) | 73.4 (72.7; 74.2) | 580.4 (578.9; 581.9) | 6.4 (6.3; 6.6) |
| 9-11 | 13 (12.8) | 6 (5.9) | 11 (10.8) | 27 (26.5) | 45 (44.0) | | 115.6 (113.1; 118.0) | 74.4 (72.3; 76.5) | 579.2 (577.3; 581.0) | 6.4 (6.1; 6.8) |
| ≥12 | 6 (7.9) | 6 (7.9) | 6 (7.9) | 26 (34.2) | 32 (42.1) | | 110.8 (108.5; 113.0) | 72.1 (70.2; 73.9) | 578.6 (576.3; 580.8) | 6.0 (5.5; 6.6) |
| **2004-5** |  |  |  |  |  | |  |  |  |  |
| Family income|| | p<0.01* | | | | | | p=0.02** | p=0.02** | p=0.51** | p=0.61** |
| ≤1 | 9 (10.1) | 6 (6.7) | 7 (7.9) | 18 (20.2) | 49 (55.1) | | 112.6 (110.3; 114.9) | 72.1 (70.3; 73.9) | 580.6 (576.8; 584.4) | 6.4 (6.0; 6.8) |
| 1.1-3 | 43 (9.8) | 25 (5.7) | 61 (13.9) | 82 (18.6) | 229 (52.0) | | 115.6 (114.3; 116.9) | 74.5 (73.5; 75.5) | 581.3 (579.6;.583.1) | 6.5 (6.3; 6.6) |
| 3.1-6 | 42 (12.8) | 34 (10.3) | 40 (12.2) | 85 (25.8) | 128 (38.9) | | 114.4 (113.3; 115.6) | 73.7 (72.8; 74.6) | 580.1 (578.1; 582.2) | 6.4 (6.2; 6.6) |
| 6.1-10 | 17 (13.6) | 12 (9.6) | 20 (16.0) | 35 (28.0) | 41 (32.8) | | 112.5 (110.7; 114.3) | 72.4 (71.0; 73.8) | 579.9 (576.9; 583.0) | 6.3 (6.1; 6.5) |
| >10 | 5 (7.4) | 5 (7.4) | 5 (7.4) | 21 (30.9) | 32 (46.9) | | 111.9 (109.2; 114.5) | 71.6 (69.6; 73.7) | 577.5 (575.6; 579.4) | 6.6 (6.2; 7.0) |
| Schooling | p<0.01* | | | | | | p=0.56** | p=0.44** | p=0.01** | p=0.47** |
| 0-4 | 10 (10.6) | 8 (8.5) | 3 (3.2) | 16 (17.0) | 57 (60.7) | | 114.3 (112.1; 116.6) | 72.8 (71.1; 74.4) | 581.3 (576.5; 586.2) | 6.3 (6.0; 6.6) |
| 5-8 | 35 (10.2) | 21 (6.1) | 42 (12.2) | 63 (18.4) | 182 (53.1) | | 114.9 (113.4; 116.4) | 73.8 (72.7; 74.9) | 582.6 (580.5; 584.6) | 6.5 (6.4; 6.7) |
| 9-11 | 59 (11.1) | 42 (7.9) | 75 (14.2) | 139 (26.2) | 215 (40.6) | | 114.3 (113.3; 115.2) | 73.8 (73.1; 74.6) | 579.7 (578.2; 581.1) | 6.4 (6.3; 6.5) |
| ≥12 | 12 (14.3) | 11 (13.1) | 13 (15.5) | 23 (27.4) | 25 (29.7) | | 112.8 (110.3; 115.3) | 72.4 (70.5; 74.4) | 576.4 (574.2; 578.5) | 6.4 (6.0; 6.7) |
| Asset index^¶^ | p=0.02* | | | | | | p=0.49** | p=0.77** | p=0.18** | p=0.92** |
| D/E (poorest) | 28 (8.4) | 25 (7.5) | 44 (13.2) | 60 (18.0) | 176 (52.9) | | 114.5 (113.2; 115.9) | 73.8 (72.8; 74.8) | 581.0 (579.2; 582.8) | 6.4 (6.2; 6.5) |
| C | 52 (13.4) | 25 (6.4) | 46 (11.8) | 100 (25.7) | 166 (42.7) | | 114.7 (113.4; 116.0) | 73.7 (72.7; 74.6) | 579.9 (578.2; 581.7) | 6.4 (6.3; 6.6) |
| A/B (richest) | 13 (7.9) | 13 (7.9) | 26 (15.8) | 45 (27.3) | 68 (41.1) | | 113.4 (111.8; 115.0) | 73.1 (71.9; 74.4) | 578.2 (576.7; 579.8) | 6.4 (6.2; 6.7) |
| Energy intake (quintiles) | p=0.47* | | | | | | p=0.31** | p=0.29** | p=0.20** | p=0.80** |
| First | 32 (15.2) | 13 (6.2) | 25 (11.9) | 47 (22.3) | 94 (44.4) | | 115.2 (113.6; 116.9) | 74.6 (73.4; 75.8) | 580.7 (579.0; 582.5) | 6.5 (6.3; 6.8) |
| Second | 23 (11.0) | 16 (7.6) | 31 (14.8) | 42 (20.0) | 98 (46.6) | | 114.2 (112.6; 115.8) | 73.7 (72.5; 74.9) | 579.0 (577.0; 581.1) | 6.4 (6.2; 6.6) |
| Third | 21 (10.0) | 17 (8.1) | 35 (16.7) | 51 (24.3) | 86 (40.9) | | 113.4 (111.8; 115.0) | 72.7 (71.4; 74.0) | 579.0 (576.7; 581.2) | 6.4 (6.2; 6.6) |
| Fourth | 19 (9.1) | 18 (8.6) | 21 (10.0) | 56 (26.7) | 96 (45.6) | | 113.6 (112.1; 115.0) | 73.2 (72.0; 74.3) | 581.3 (578.1; 584.5) | 6.4 (6.1; 6.6) |
| Fifth | 21 (10.0) | 18 (8.6) | 21 (10.0) | 45 (21.4) | 105 (50.0) | | 115.4 (113.4; 117.4) | 73.9 (72.5; 75.2) | 582.5 (579.7; 585.4) | 6.4 (6.2; 6.6) |
| Leisure physical activity (≥150min) | p=0.68* | | | | | | p=0.32** | p=0.94** | p=0.74** | p=0.87** |
| No | 100 (11.1) | 73 (8.1) | 114 (12.7) | 200 (22.2) | 413 (45.9) | | 114.2 (113.4; 115.0) | 73.6 (73.0; 74.2) | 580.5 (579.3; 581.8) | 6.4 (6.3; 6.5) |
| Yes | 16 (10.6) | 9 (6.0) | 19 (12.6) | 41 (27.2) | 66 (43.6) | | 115.3 (113.6; 116.9) | 73.7 (72.3; 75.1) | 580.0 (578.2; 581.9) | 6.4 (6.2; 6.7) |
| Alcohol consumption | p=0.23* | | | | | | p=0.80** | p=0.49** | p=0.44** | p=0.59** |
| No | 43 (9.9) | 26 (6.0) | 54 (12.5) | 110 (25.4) | 200 (46.2) | | 114.2 (113.0; 115.4) | 75.3 (74.7; 75.8) | 580.0 (578.5; 581.5) | 6.5 (6.3; 6.6) |
| Yes | 72 (11.9) | 54 (8.9) | 77 (12.7) | 129 (21.3) | 274 (45.2) | | 114.4 (113.5; 115.3) | 75.5 (75.1; 75.9) | 580.9 (579.3; 582.4) | 6.4 (6.3; 6.5) |
| Current smoking | p=0.82* | | | | | | p<0.01** | p<0.01** | p=0.41** | p=0.61** |
| No | 81 (10.8) | 56 (7.5) | 100 (13.4) | 173 (23.1) | 339 (45.2) | | 115.0 (114.1; 115.9) | 75.7 (75.4; 76.1) | 580.2 (579.0; 581.4) | 6.4 (6.3; 6.5) |
| Yes | 35 (11.6) | 26 (8.6) | 33 (10.9) | 68 (22.5) | 140 (46.4) | | 112.8 (111.4; 114.1) | 74.5 (73.8; 75.1) | 581.2 (578.8; 583.6) | 6.5 (6.3; 6.6) |
| Body mass index (kg/m^2^) | p=0.65* | | | | | | p<0.01** | p<0.01** | p<0.01 | p<0.01 |
| <25.0 | 71 (10.6) | 59 (8.8) | 89 (13.3) | 151 (22.6) | 298 (44.6) | | 112.3 (111.5; 113.1) | 72.0 (71.3; 72.6) | 578.2 (577.2; 579.3) | 6.3 (6.2; 6.4) |
| 25.0-29.9 | 20 (10.6) | 10 (5.3) | 25 (13.3) | 46 (24.5) | 87 (46.3) | | 116.5 (115.0; 118.0) | 75.9 (74.8; 77.0) | 584.9 (581.2; 588.5) | 6.7 (6.4; 6.9) |
| ≥30.0 | 16 (16.5) | 8 (8.3) | 14 (14.4) | 20 (20.6) | 39 (40.2) | | 124.9 (121.2; 128.6) | 80.7 (78.3; 83.2) | 591.2 (585.9; 596.4) | 6.9 (6.5; 7.3) |
| ||Minimum wages. ¶Brazilian Association of Research Companies. *Chi-square. **Analysis of variance. | | | | | | | | | | |
