# Supplementary material for: Breastfeeding and maternal cardiovascular risk factors: 1982 Pelotas Birth Cohort
Source: Sci Rep. 2019 Sep 11;9:13092. doi: 10.1038/s41598-019-49576-1 (PMC6739402; doi:10.1038/s41598-019-49576-1)
Supplement: Supplementary file 2 — Supplementary Table S2. Glycaemia and lipid profile according to biological, socioeconomic and behavioral variables (n=1136). Pelotas, 1982–2012. [file 41598_2019_49576_MOESM2_ESM.doc]

**Breastfeeding and maternal cardiovascular risk factors: 1982 Pelotas Birth Cohort**

**Authors**

Natália P. Lima

Diego G. Bassani

Elma Izze S. Magalhães

Fernando C. Barros

Bernardo L. Horta

| **Supplementary Table S2.** Glycaemia and lipid profile according to biological, socioeconomic and behavioral variables (n=1136). Pelotas, 1982-2012. | | | | | | |
| --- | --- | --- | --- | --- | --- | --- |
| Variables | Glycaemia  (mg/dl) | Cholesterol  (mg/dl) | LDL  (mg/dl) | HDL  (mg/dl) | Non-HDL  (mg/dl) | Triglycerides#  (mg/dl) |
|  | Mean (CI95%) | Mean (CI95%) | Mean (CI95%) | Mean (CI95%) | Mean (CI95%) | Mean (CI95%) |
| **Birth** |  |  |  |  |  |  |
| European ancestry (quintiles) | p=0.85 | p=0.11 | p=0.09 | p=0.89 | p=0.04 | p<0.01 |
| First | 85.8 (82.1; 89.4) | 181.8 (176.7; 186.9) | 103.3 (99.1; 107.4) | 61.0 (59.3; 62.7) | 120.8 (116.2; 125.4) | 78.0 (73.4; 83.0) |
| Second | 87.4 (85.2; 89.6) | 191.3 (185.8; 196.8) | 111.3 (106.6; 115.9) | 60.5 (58.7; 62.4) | 130.8 (125.6; 135.9) | 92.1 (85.6; 99.1) |
| Third | 87.8 (84.9; 90.7) | 189.1 (184.1; 194.1) | 108.0 (104.1; 111.9) | 60.2 (58.4; 62.0) | 128.9 (124.0; 133.8) | 96.6 (89.5; 104.3) |
| Fourth | 88.3 (84.3; 92.3) | 186.2 (181.3; 191.2) | 106.0 (102.2; 109.8) | 60.6 (58.6; 62.5) | 125.7 (121.5; 129.9) | 91.7 (85.5; 98.4) |
| Fifth | 86.9 (84.1; 89.6) | 187.5 (182.7; 192.4) | 106.3 (102.5; 110.2) | 61.5 (59.6; 63.3) | 126.1 (121.6; 120.6) | 92.5 (86.0; 99.4) |
| Family income|| | p=0.38 | p=0.16 | p=0.10 | p=0.07 | p=0.11 | p=0.04 |
| ≤1 | 88.4 (85.3; 91.5) | 184.5 (180.5; 188.6) | 104.7 (101.5; 107.9) | 60.3 (58.8; 61.9) | 124.2 (120.3; 128.1) | 87.3 (82.1; 92.7) |
| 1.1-3 | 86.5 (85.2; 87.9) | 187.4 (184.6; 190.2) | 107.9 (105.7; 110.2) | 59.8 (58.8; 60.8) | 127.6 (125.0; 130.1) | 91.6 (88.1; 95.2) |
| 3.1-6 | 89.6 (84.1; 95.0) | 191.3 (185.4; 197.3) | 108.8 (104.1; 113.4) | 62.9 (60.9; 64.9) | 128.4 (123.2; 133.7) | 93.4 (86.5; 100.9) |
| 6.1-10 | 84.2 (80.2; 88.1) | 185.6 (174.1; 197.2) | 105.6 (96.7; 114.5) | 61.5 (57.4; 65.6) | 124.1 (113.5; 134.7) | 84.0 (71.2; 99.3) |
| >10 | 83.2 (77.6; 88.9) | 172.9 (160.1; 185.6) | 92.4 (80.7; 104.0) | 63.6 (58.3; 69.0) | 109.2 (96.5; 121.9) | 66.6 (54.7; 80.9) |
| Maternal schooling | p=0.30 | p=0.27 | p=0.24 | p<0.01 | p=0.33 | p=0.47 |
| 0-4 | 87.1 (85.1; 89.1) | 184.6 (181.5; 187.8) | 105.9 (103.3; 108.4) | 59.5 (58.4; 60.7) | 125.1 (122.1; 128.1) | 88.3 (84.3; 92.4) |
| 5-8 | 88.5 (86.1; 90.8) | 188.5 (185.2; 191.7) | 108.6 (106.0; 111.2) | 60.3 (59.2; 61.4) | 128.2 (125.3; 131.1) | 91.4 (87.4; 95.5) |
| 9-11 | 84.8 (82.8; 86.9) | 190.3 (182.9; 197.8) | 107.8 (102.0; 113.7) | 62.0 (59.5; 64.4) | 128.4 (121.7; 135.0) | 94.8 (86.5; 104.0) |
| ≥12 | 84.5 (81.6; 87.3) | 189.0 (181.3; 196.8) | 102.5 (96.5; 108.5) | 66.3 (63.0; 69.7) | 122.7 (115.3; 130.0) | 86.9 (76.9; 98.4) |
| **2004-5** |  |  |  |  |  |  |
| Family income|| | p=0.96 | p=0.44 | p=0.89 | p<0.01 | p=0.97 | p=0.91 |
| ≤1 | 87.9 (84.2; 91.7) | 183.3 (175.9; 190.6) | 104.7 (99.4; 109.9) | 57.0 (54.4; 59.7) | 126.2 (118.6; 133.8) | 90.1 (80.0; 101.5) |
| 1.1-3 | 87.5 (85.0; 90.0) | 185.1 (181.7; 188.5) | 106.6 (104.0; 109.3) | 59.1 (57.9; 60.3) | 126.0 (122.9; 129.1) | 89.1 (85.0; 93.4) |
| 3.1-6 | 87.3 (84.9; 89.6) | 188.8 (184.9; 192.8) | 108.0 (104.7; 111.4) | 61.5 (60.1; 62.9) | 127.3 (123.7; 131.0) | 90.5 (85.7; 95.6) |
| 6.1-10 | 86.2 (82.8; 89.7) | 188.8 (182.7; 194.9) | 106.4 (101.3; 111.6) | 62.8 (60.6; 65.1) | 126.0 (120.3; 131.7) | 93.2 (85.5; 101.5) |
| >10 | 85.9 (83.1; 88.7) | 189.8 (181.3; 198.4) | 106.1 (99.3; 112.9) | 65.1 (62.3; 68.0) | 124.7 (117.0; 132.4) | 87.5 (78.2; 97.9) |
| Schooling | p=0.56 | p<0.01 | p=0.06 | p<0.01 | p=0.15 | p=0.56 |
| 0-4 | 87.8 (82.4; 93.2) | 180.0 (173.3; 186.7) | 106.5 (100.9; 112.1) | 55.3 (53.0; 57.7) | 124.7 (118.6; 130.7) | 84.6 (77.1; 92.9) |
| 5-8 | 87.8 (85.5; 90.1) | 183.6 (179.7; 187.5) | 105.4 (102.3; 108.5) | 58.1 (56.7; 59.4) | 125.5 (121.8; 129.2) | 91.6 (86.5; 97.0) |
| 9-11 | 87.2 (85.2; 89.3) | 190.4 (187.4; 193.4) | 108.9 (106.4; 111.4) | 62.2 (61.1; 63.3) | 128.2 (125.4; 131.0) | 90.4 (86.7; 94.2) |
| ≥12 | 83.9 (81.6; 86.2) | 185. 4 (178.0; 192.8) | 100.5 (94.7; 106.2) | 65.6 (62.6; 68.5) | 119.8 (113.2; 126.5) | 87.5 (78.3; 97.8) |
| Asset index¶ | p=0.79 | p<0.01 | p=0.07 | p<0.01 | p=0.05 | p=0.08 |
| D/E (poorest) | 87.2 (84.6; 89.8) | 181.5 (178.0; 185.1) | 104.4 (101.5; 107.3) | 58.2 (57.0; 59.5) | 123.3 (119.9; 126.7) | 86.7 (82.4; 91.3) |
| C | 87.8 (85.2; 90.5) | 189.9 (186.2; 193.6) | 109.1 (106.1; 112.2) | 60.8 (59.5; 62.1) | 129.1 (125.6; 132.5) | 93.3 (88.6; 98.3) |
| A/B (richest) | 86.3 (84.3; 88.3) | 187.0 (181.4; 192.7) | 105.0 (100.4; 109.6) | 63.3 (61.4; 65.3) | 123.7 (118.6; 128.8) | 86.1 (79.9; 92.8) |
| Energy intake (quintiles) | p=0.99 | p=0.55 | p=0.33 | p=0.09 | p=0.53 | p=0.58 |
| First | 87.1 (84.5; 89.7) | 187.1 (181.9; 192.3) | 106.8 (102.8; 110.8) | 59.6 (57.9; 61.4) | 127.5 (122.5; 132.4) | 92.7 (86.0; 100.0) |
| Second | 87.1 (83.4; 90.8) | 188.1 (183.4; 192.8) | 106.0 (102.4; 109.6) | 62.4 (60.5; 64.2) | 125.7 (121.6; 129.9) | 90.5 (84.5; 96.8) |
| Third | 87.7 (83.8; 91.5) | 187.7 (182.7; 192.7) | 107.1 (102.8; 111.3) | 61.3 (59.6; 63.0) | 126.4 (121.8; 131.0) | 91.4 (85.7; 97.5) |
| Fourth | 87.6 (84.9; 90.3) | 188.3 (183.5; 193.0) | 110.1 (106.1; 114.1) | 59.5 (57.9; 61.2) | 128.8 (124.3; 133.2) | 89.8 (84.1; 95.9) |
| Fifth | 86.5 (84.2; 88.9) | 183.1 (178.5; 187.7) | 104.2 (100.5; 108.0) | 59.8 (58.1; 61.5) | 123.3 (118.9; 127.7) | 85.8 (80.0; 92.0) |
| Leisure physical activity (≥150min) | p=0.97 | p=0.93 | p=0.77 | p=0.24 | p=0.58 | p=0.49 |
| No | 87.2 (85.7; 88.8) | 186.9 (184.5; 189.3) | 107.0 (105.0; 108.9) | 60.3 (59.5; 61.2) | 126.6 (124.4; 128.8) | 90.4 (87.4; 93.5) |
| Yes | 87.1 (84.1; 90.2) | 186.6 (181.5; 191.8) | 106.2 (102.0; 110.4) | 61.7 (59.6; 63.8) | 125.0 (120.3; 129.7) | 87.7 (81.5; 94.3) |
| Alcohol consumption | p=0.69 | p=0.53 | p=0.68 | p=0.03 | p=0.87 | p=0.55 |
| No | 86.9 (84.9; 88.8) | 186.0 (182.5; 189.5) | 106.3 (103.6; 109.0) | 59.5 (58.3; 60.8) | 126.4 (123.3; 129.6) | 91.0 (86.7; 95.5) |
| Yes | 87.4 (85.5; 89.4) | 187.4 (184.6; 190.2) | 107.1 (104.8; 109.4) | 61.3 (60.3; 62.3) | 126.1 (123.5; 128.8) | 89.3 (85.8; 92.9) |
| Current smoking | p=0.06 | p=0.17 | p=0.51 | p>0.01 | p=0.64 | p=0.69 |
| No | 88.0 (86.3; 89.8) | 187.8 (185.3; 190.4) | 106.5 (104.5; 108.5) | 61.8 (60.9; 62.7) | 126.0 (123.7; 128.4) | 90.4 (87.2; 93.7) |
| Yes | 85.1 (82.9; 87.3) | 184.4 (180.2; 188.6) | 107.8 (104.2; 111.3) | 57.3 (55.8; 58.8) | 127.1 (123.1; 131.1) | 89.1 (84.1; 94.3) |
| Body mass index (kg/m^2^) | p<0.01 | p<0.01 | p<0.01 | p=0.02 | p<0.01 | p<0.01 |
| <25.0 | 85.8 (84.2; 87.3) | 184.9 (182.2; 187.5) | 105.2 (103.0; 107.3) | 61.3 (60.4; 62.3) | 123.5 (121.1; 125.9) | 96.5 (92.4; 100.6) |
| 25.0-29.9 | 86.8 (84.3; 89.3) | 192.3 (187.1; 197.5) | 110.9 (106.6; 115.2) | 60.6 (58.7; 62.5) | 131.8 (126.9; 136.6) | 109.0 (100.5; 117.5) |
| ≥30.0 | 100.1 (91.3; 108.8) | 200.0 (192.1; 207.8) | 117.0 (111.2; 122.9) | 57.4 (55.0; 59.7) | 142.6 (134.8; 150.5) | 147.5 (124.0; 171.1) |
| #Geometric mean. ||Minimum wages. ¶Brazilian Association of Research Companies. Analysis of variance. | | | | | | |
